# Supplementary material for: Short-term radiofrequency exposure from new generation mobile phones reduces EEG alpha power with no effects on cognitive performance
Source: Sci Rep. 2018 Dec 20;8:18010. doi: 10.1038/s41598-018-36353-9 (PMC6301959; doi:10.1038/s41598-018-36353-9)
Supplement: Supplementary file 1 — Supplementary Materials [file 41598_2018_36353_MOESM1_ESM.docx]

# Short-term radiofrequency exposure from new generation mobile phones reduces EEG alpha power with no effects on cognitive performance

# Supplementary Material

Zsuzsanna Vecsei, Balázs Knakker, Péter Juhász, György Thuróczy, Attila Trunk, István Hernádi^CA^

**Corresponding author’s (CA) address:**

University of Pécs, Department of Experimental Zoology and Neurobiology

Ifjúság útja 6, H-7624, Pécs, Hungary

*Phone*: +36 72 503607, *Fax*: +36 72 501517

*E-mail*: hernadi.istvan@pte.hu

# Supplementary Material 1: Exposure Systems and RF Dosimetry

## Introduction

The exact conditions of exposure go beyond the scope of the methods in the main text. Hence, we elaborate on this topic in the first section. The RF exposure system consisted of signal sources (mobile phone or signal generator), power amplifiers, the antenna holding fixture and the antenna itself. Fig. 3 in the main text shows a block diagram of the setup.

## Signal Source and Amplification

### UMTS signal

The UMTS RF exposure was administered by means of a standard Nokia 6650 (Nokia, Espoo, Finland) MP via Phoenix Service Software (v. 2005/44_4_120; Nokia, Espoo, Finland) for 20 minutes. The MP was connected to RF amplifier (Bonn Hungary Electronics Ltd., Hungary). The patch antenna was connected to the output of RF amplifier. For the details on the exposure device and conditions, see Vecsei *et al.*^1^

### LTE signal

The LTE “signal cocktail” was generated by Anritsu MG3700A (Anritsu Co., Japan) programmable signal generator. This device is able to generate an arbitrary baseband waveform and mix it on a RF carrier of arbitrary frequency and power. The specific LTE signal used in the experiment was created on a PC with Anritsu's software (IQproducer, Version 7.00, Anritsu Corporation) then uploaded to the signal generator. During the experiment itself the signal generator was remote controlled via TCP/IP by our custom-developed driver software. We selected the 16QAM modulation scheme and all 100 resource blocks possible in this configuration were active. It should be noted that such signals rarely occur in practice however from an experimental standpoint it approximates well the "worst case", that is, the one causing the highest possible exposition. The signal generator was connected to RF power amplifier BPAM14 (Bonn Hungary Electronics, Hungary), capable of operating between 1700-2000 MHz, with a maximum output power of +41 dBm. The same patch antenna was used as in the UMTS experiment. Our previous measurements revealed that the SAR pattern generated by the patch antenna was localized close to the area of the antenna surface and was highly concentrated there. In our experiment we set the maximum SAR to be 1.8 W/kg and distance between the ear and antenna to be 7 mm. These values determine the input power to be 2.72 W (34.35 dBm).

## Patch antenna

The exposure device was based on a single-sided round dual band patch antenna (Reinheimer Elektronik, Wettenberg, Germany; model no: M30EXO-0250-XX) with resonant surface and ground plane on the same side. This antenna arrangement provided sufficiently enhanced local exposure at the ear region. The 31-mm-diameter 0.5-mm thick patch antenna was encapsulated in a 40-mm-diameter 7-mm-thick transparent plastic capsule with Styrofoam isolation. The antenna was tuned with a waveguide resistor resonator and capacitors (SMD realization). Reflection measurements demonstrated that the capsule did not have any significant effect on the antenna's reflection parameters or effectiveness. The non-metallic side of the antenna was designated to be the application side. The covering case on this side was 1 mm thick. The antenna described here was developed earlier and was used in earlier investigations already published.^2-4^ A plastic holding fixture with spherical joints allowed precise, repeatable antenna positioning. The patch antenna was mounted in a position mimicking the normal use of an MP, the centre of the patch antenna was near the exit of the ear canal, above the tragus, at a distance of 7 mm. The EEG cap compressed the pinna, allowing more precise positioning.

## Measurement of specific absorption rate (SAR)

In any experiment dealing with the possible biological effects of RF exposure the accurate quantification of the SAR is of critical importance. Unfortunately, SAR is not directly measurable; in practice it is either derived from numerical simulation or it is calculated from electric field measurements made in a phantom filled with brain tissue-equivalent liquid. The measurements of absorbed radiofrequency power in the head, termed specific absorption rate (SAR, W/kg), were made in a standard human head SAM phantom (Specific Anthropomorphic Mannequin phantom, Antennessa, France) filled with standard brain tissue-equivalent liquid (Satimo, France) according to CENELEC standard EN 50361 (CENELEC, 2002). A small electric field probe (O6-EP64, Satimo, France) connected to a microvoltmeter (Keithley, USA) was used for the measurement of the electric field strength within the liquid. The liquid calibration was based on an open-ended coaxial cable immersed in the phantom liquid connected to a vector network analyser (Wiltron 360B, USA). The electric field probe motion was realized by a servo-driven XYZ positioning system with a 3D step motor robot (Charlyrobot SA, France).

# Supplementary Material 2: Stroop Test Quality Control

With respect to our own implementation of the Stroop test, we evaluated whether the FAC and IF to be measured could be reliably detected, so as to enable the measurement of RT changes caused by the RF exposure. You can find the description of the quality control analyses below.

For Stroop test quality control mixed ANOVA was used in a three-way (2 × 2 × 3) × 2 design considering only RTs of sham exposed sessions. Within-subject factors as time (two levels: pre-exposure, post-exposure) × task (two levels: word-naming (WN), colour-naming (CN)) and condition (three levels: congruent, neutral, incongruent) and between subject factor RF type (two levels: UMTS, LTE) were considered at α-level of 0.05 with adjustment for multiple comparisons (Tukey correction) at post hoc tests. Interference (IF) and facilitation (FAC) effects (see main text for definitions) were tested as planned comparisons and results of t-tests and mean differences (MD) are reported. Data met the assumption of normality. In the few cases when sphericity (Mauchly's Test of Sphericity) were violated a Greenhouse-Geisser correction was used and degrees of freedom are reported with one decimal digit. In descriptive statistics, groups are represented in mean RTs with standard deviation.

A significant main effect of time was found (F_1,48_ = 36.289; p < 0.001, η^2^_p_ = 0.435). At the second Stroop-test of a session participants produced significantly shorter reactions (post-exposure 674 ± 80 ms) than at the first one (pre-exposure 720 ± 88 ms) suggesting that there is an effect of practice.

A significant main effect of task was found (F_1,48_ = 41.608; p < 0.001, η^2^_p_ = 0.464). To achieve colour-naming took longer time (728 ± 99 ms) than word-naming (673 ± 76 ms), as suppressing automatic word reading to establish colour extends the processing speed which was the first finding of Stroop.^5^

A significant main effect of condition was present (F_1.4,65.9_ = 164,698; p < 0.001, η^2^_p_ = 0.774). According to post hoc test, all pairwise comparisons expressed significant differences. In the congruent condition – where words matched colours – RT was significantly shorter (655 ± 80 ms) than in the neutral (682 ± 74 ms), suggesting that FAC is present (t = -2.96, p = 0.011, MD = -25.8 ms). In the incongruent condition – where words and colours differed – RT was significantly longer (787 ± 11 ms) than in the neutral condition (682 ± 74 ms), suggesting that IF is present (t = 14.03, p < 0.001, MD = 122.3 ms).

There was a significant task × condition interaction (F_1.5,70.5_ =41.616; p < 0.001, η^2^_p_ = 0.464). According to post hoc tests, both in the CN task and in the WN task, incongruent condition differed from both the congruent and from the neutral condition (for all p < 0.001). The interaction was driven by a greater difference between conditions in CN task (15.30 < t < 18.18) than in the WN task (1.49 < t < 6.90).

There was no main effect of RF type (F_1,48_ = 0.084; p = 0.773, η^2^_p_ = 0.002), and all the interactions involving this factor were non-significant (F < 1.64; p > 0.05, η^2^_p_ < 0.04 in all cases).

To sum up, looking at the data from Sham sessions from both the CN and the WN task (the latter is not analysed in the main text), we observed that the effects expected from a well-functioning Stroop test based on the literature were consistently and reliably present, independently of RF type group assignment. Thus, we could have potentially measured cognitive effects of RF exposure in this work with a sensitivity similar to previous studies using the Stroop test to investigate cognitive impairments like dementias,^6,7^ schizophrenia^8,9^ and attention-deficit hyperactivity disorder (ADHD).^10,11^


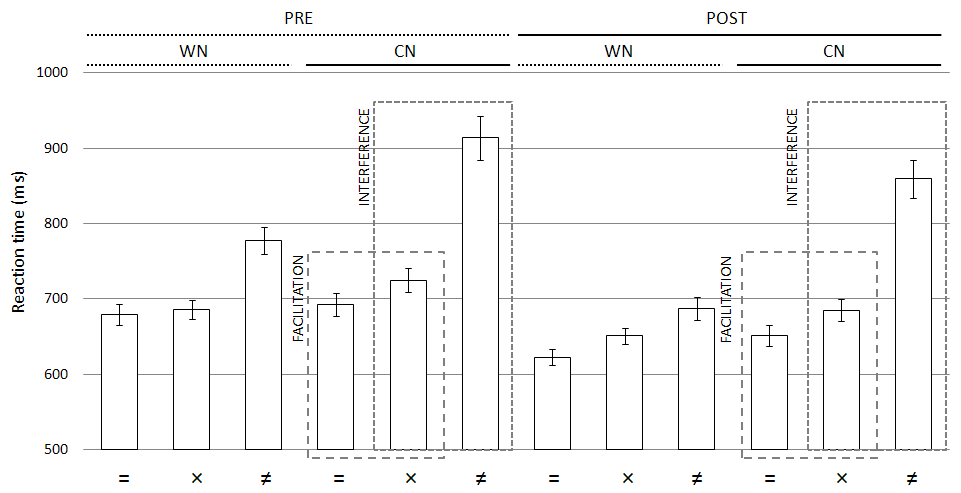


**Figure S1.** Stroop test quality control – RTs (ms) of Sham condition of both UMTS and LTE experiment. Abbreviations: Pre – before sham exposure, Post – after sham exposure, [CN] – colour-naming, [WN] – word-naming, [=] congruent, [×] neutral, [≠] incongruent.

# References

1 Vecsei, Z., Csatho, A., Thuroczy, G. & Hernadi, I. Effect of a single 30 min UMTS mobile phone-like exposure on the thermal pain threshold of young healthy volunteers. *Bioelectromagnetics* **34**, 530-541, doi:10.1002/bem.21801 (2013).

2 Trunk, A. *et al.* No effects of a single 3G UMTS mobile phone exposure on spontaneous EEG activity, ERP correlates, and automatic deviance detection. *Bioelectromagnetics* **34**, 31-42, doi:10.1002/bem.21740 (2013).

3 Parazzini, M. *et al.* Absence of short-term effects of UMTS exposure on the human auditory system. *Radiation research* **173**, 91-97, doi:10.1667/RR1870.1 (2010).

4 Stefanics, G., Thuroczy, G., Kellenyi, L. & Hernadi, I. Effects of twenty-minute 3G mobile phone irradiation on event related potential components and early gamma synchronization in auditory oddball paradigm. *Neuroscience* **157**, 453-462, doi:10.1016/j.neuroscience.2008.08.066 (2008).

5 Stroop. Studies of interference in serial verbal reactions. *Journal of Experimental Psychology,* **18**, 643-662. (1935).

6 Hutchison, K. A., Balota, D. A. & Duchek, J. M. The utility of Stroop task switching as a marker for early-stage Alzheimer's disease. *Psychology and aging* **25**, 545-559, doi:10.1037/a0018498 (2010).

7 Koss, E., Ober, B. A., Delis, D. C. & Friedland, R. P. The Stroop color-word test: indicator of dementia severity. *The International journal of neuroscience* **24**, 53-61 (1984).

8 McGrath, J., Scheldt, S., Welham, J. & Clair, A. Performance on tests sensitive to impaired executive ability in schizophrenia, mania and well controls: acute and subacute phases. *Schizophrenia research* **26**, 127-137 (1997).

9 Henik, A. & Salo, R. Schizophrenia and the stroop effect. *Behavioral and cognitive neuroscience reviews* **3**, 42-59, doi:10.1177/1534582304263252 (2004).

10 Assef, E. C., Capovilla, A. G. & Capovilla, F. C. Computerized stroop test to assess selective attention in children with attention deficit hyperactivity disorder. *The Spanish journal of psychology* **10**, 33-40 (2007).

11 Lopez-Villalobos, J. A. *et al.* [Usefulness of the Stroop test in attention deficit hyperactivity disorder]. *Revista de neurologia* **50**, 333-340 (2010).
